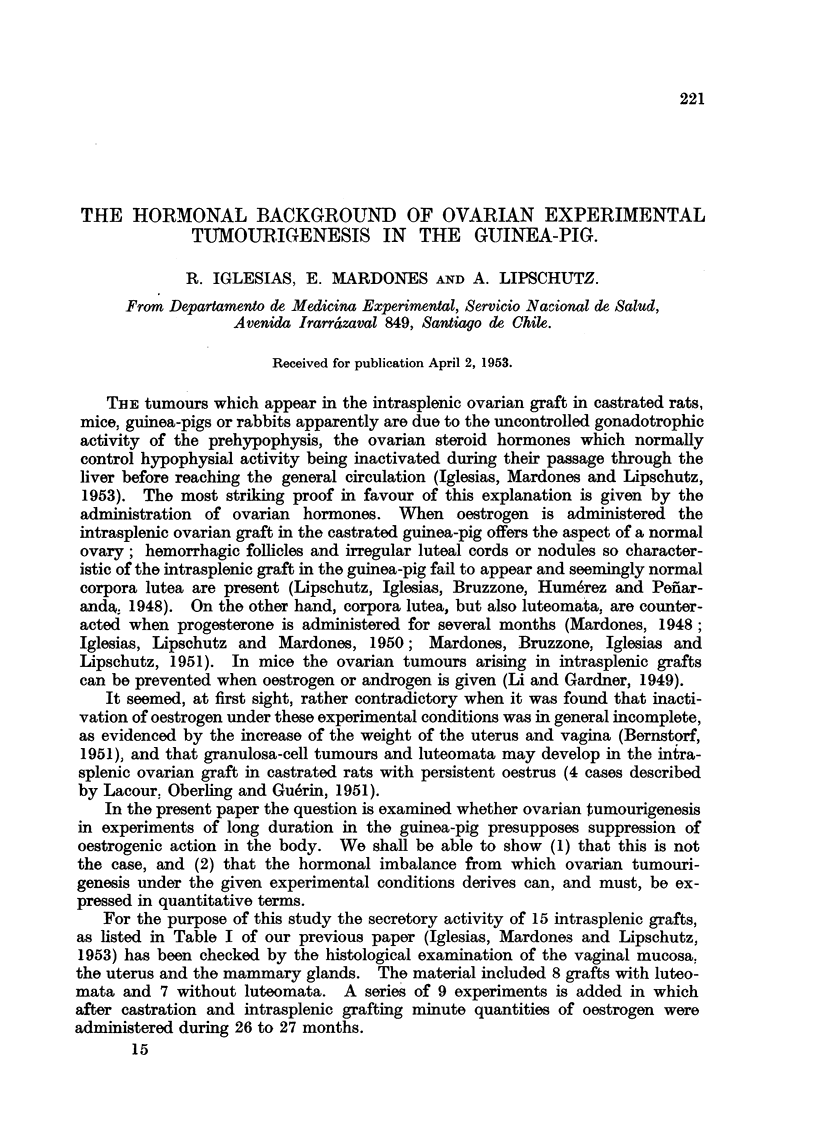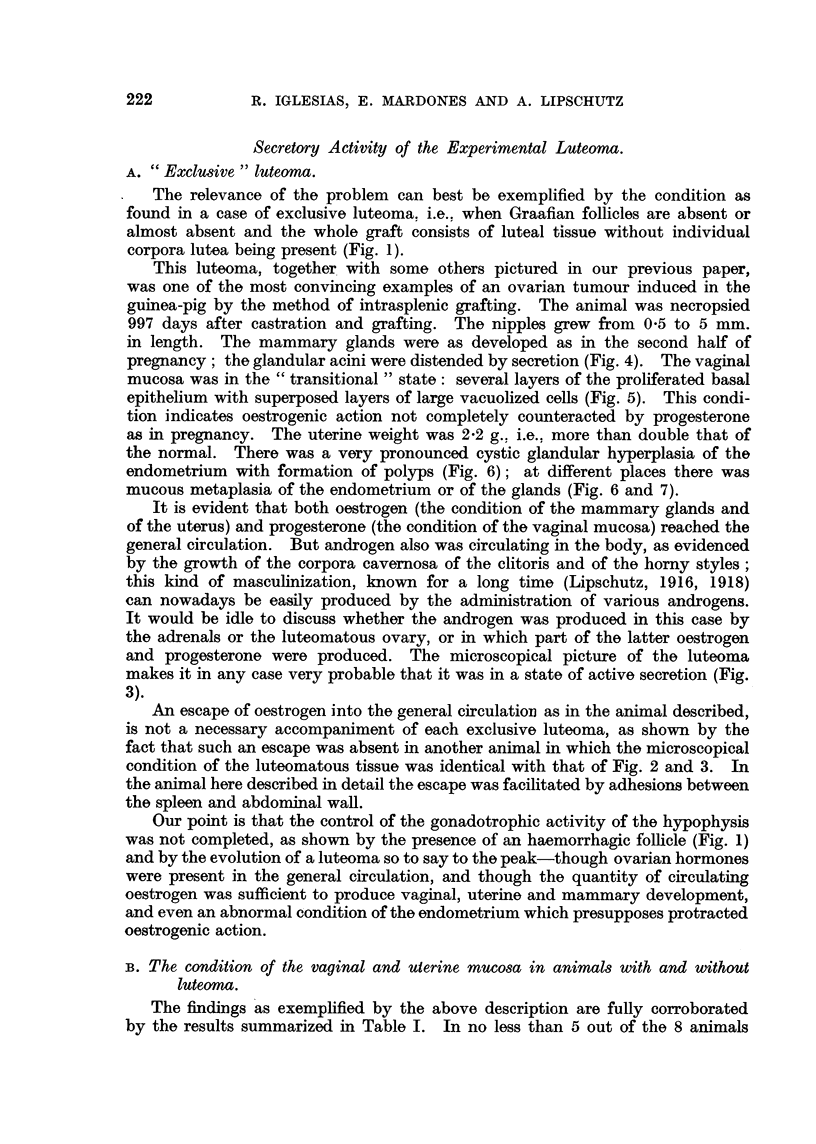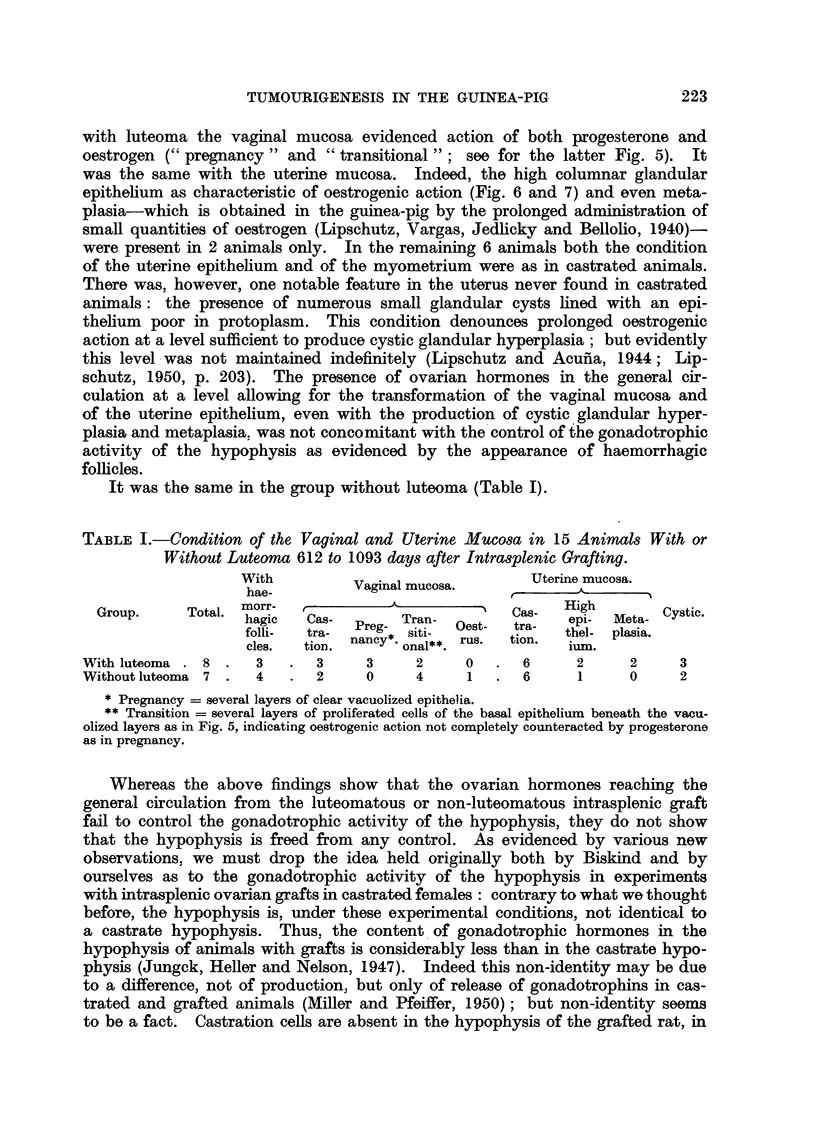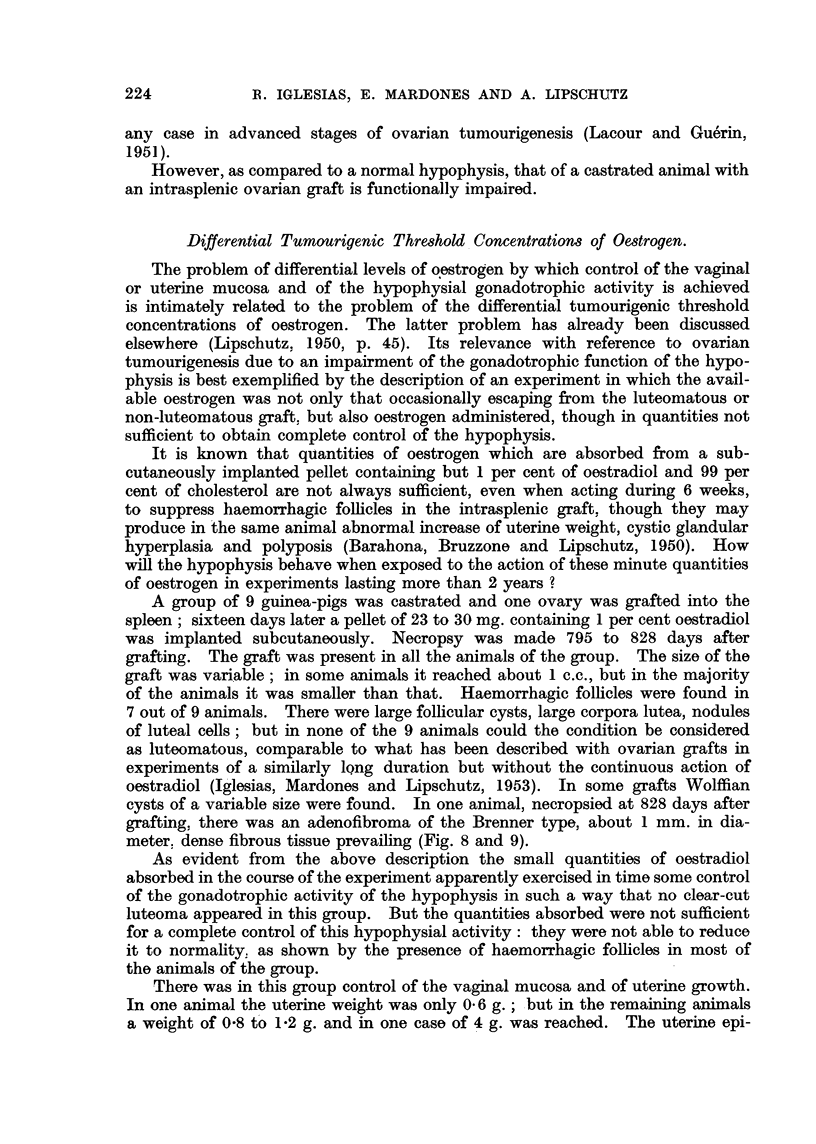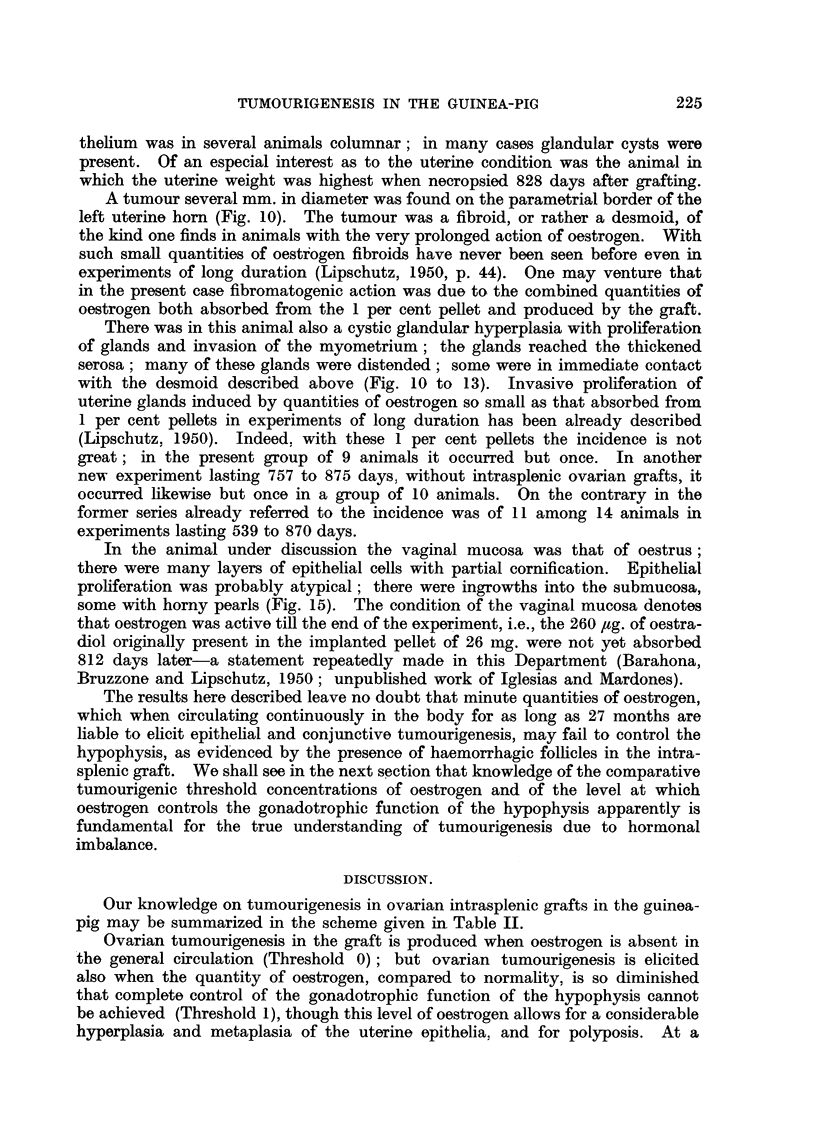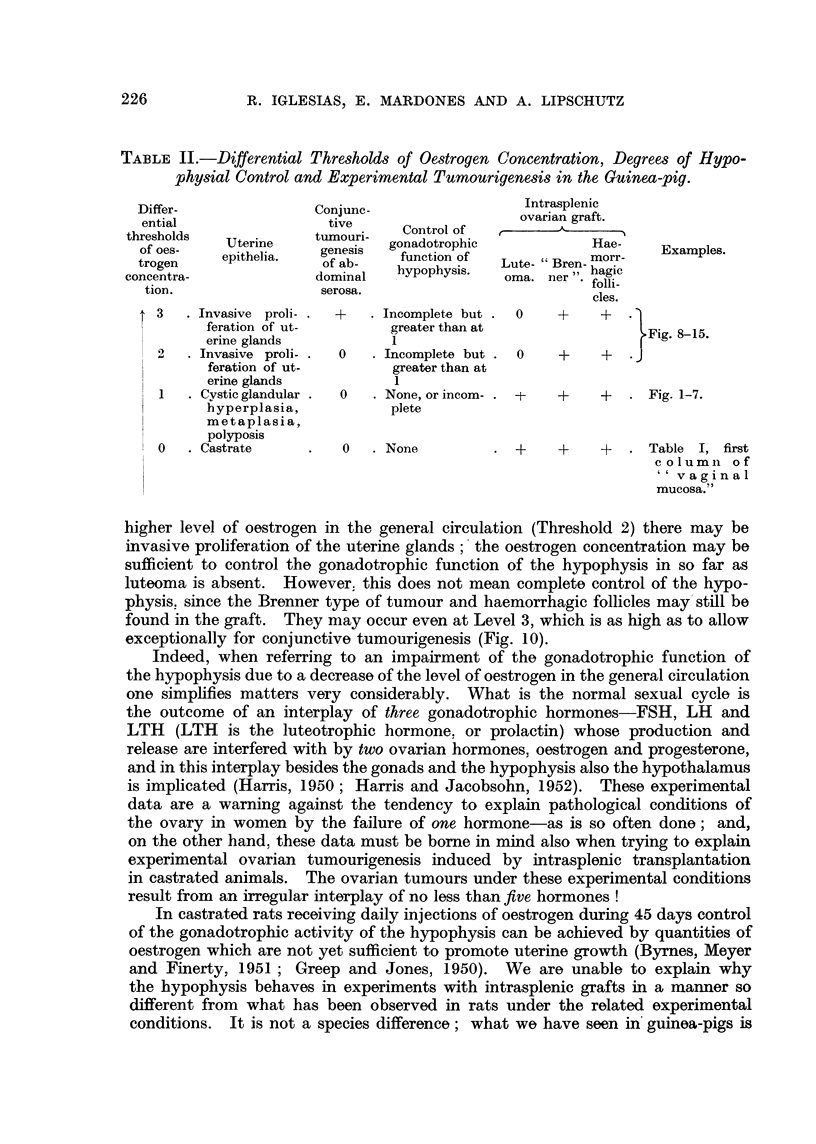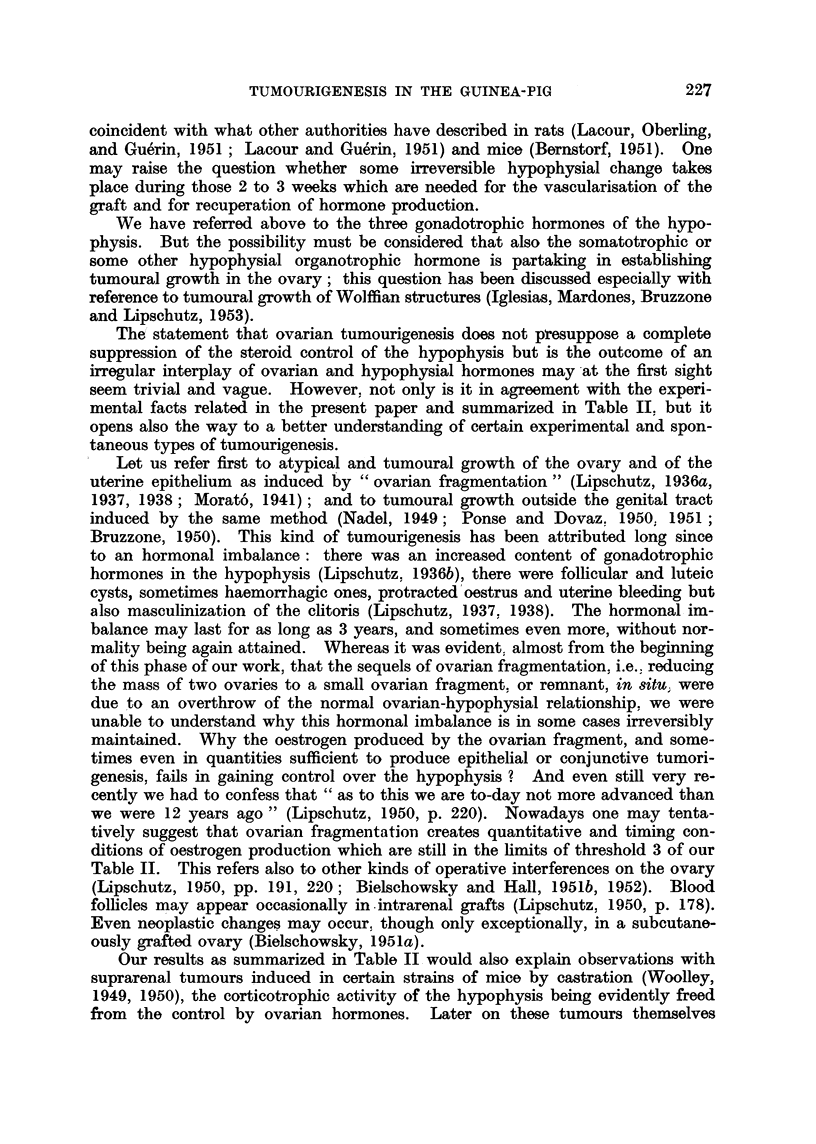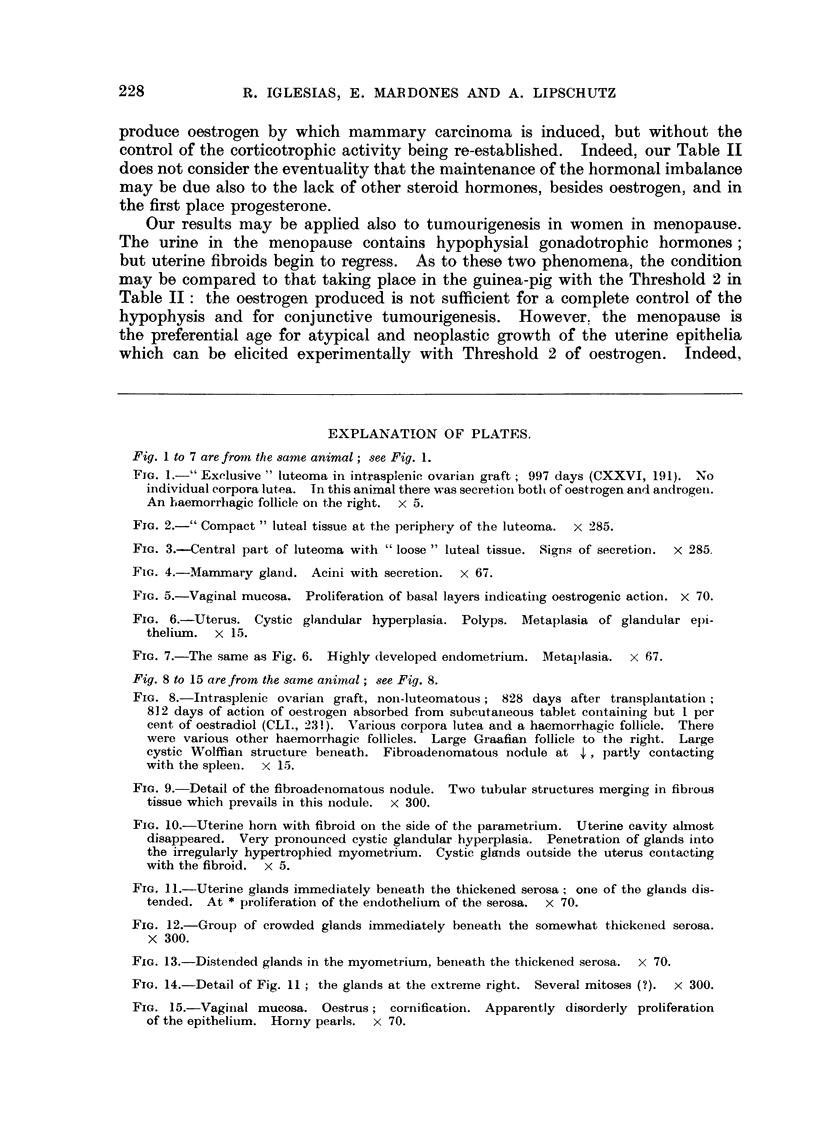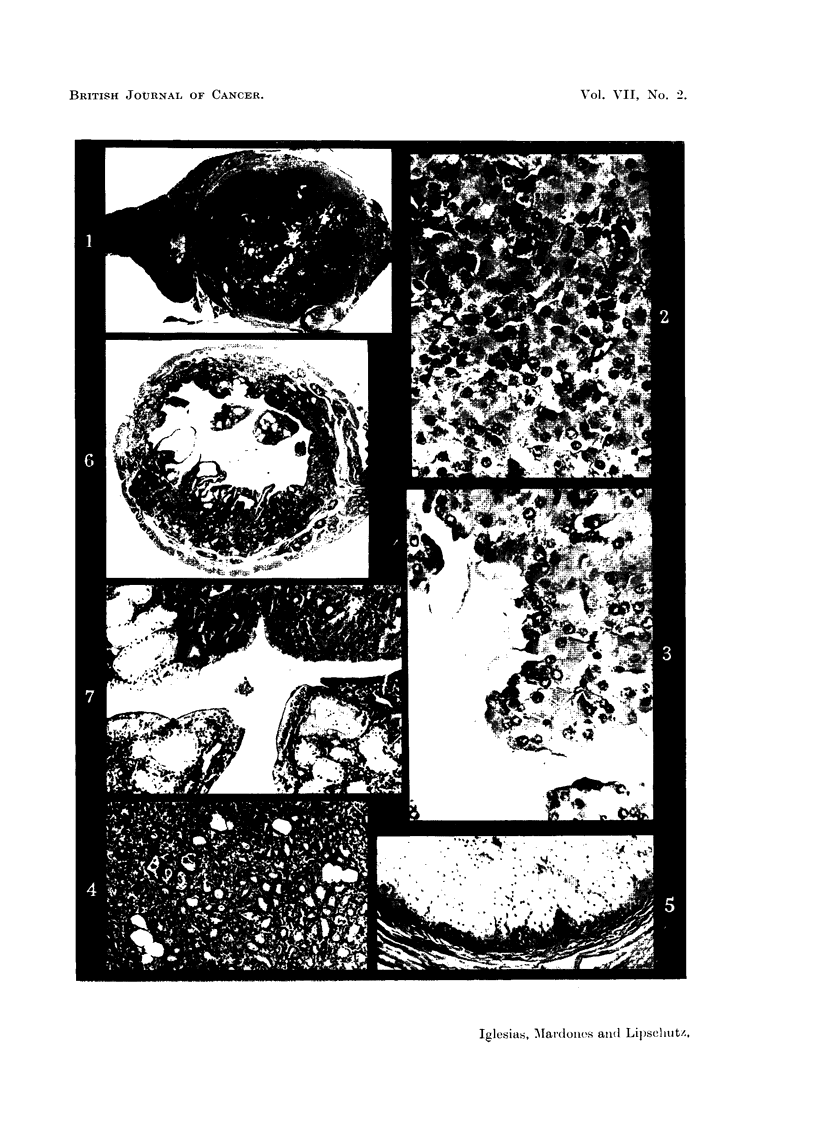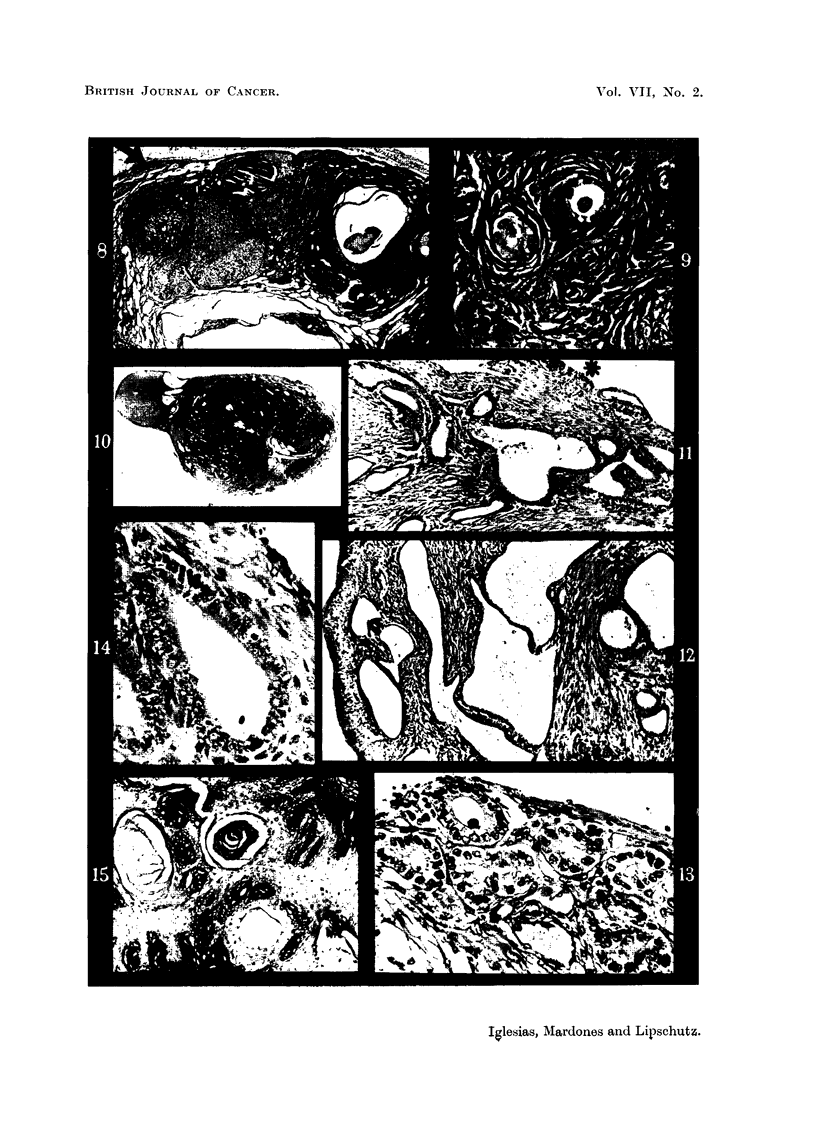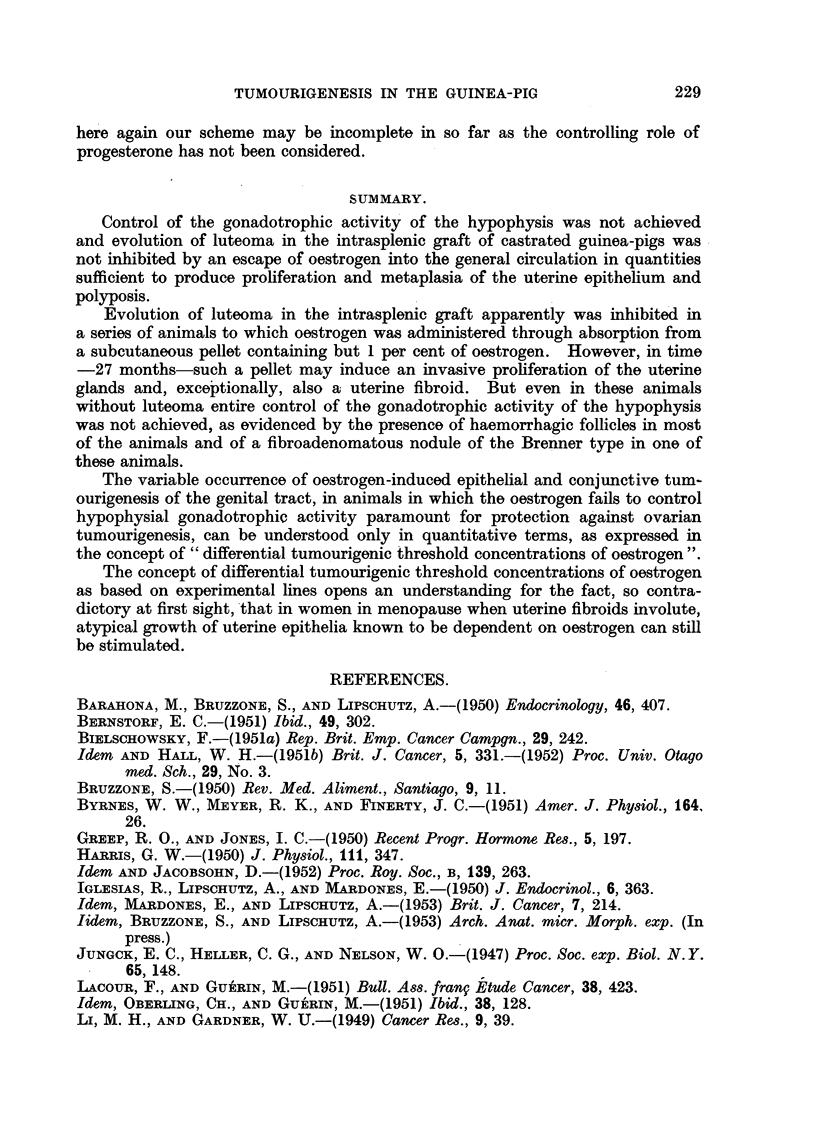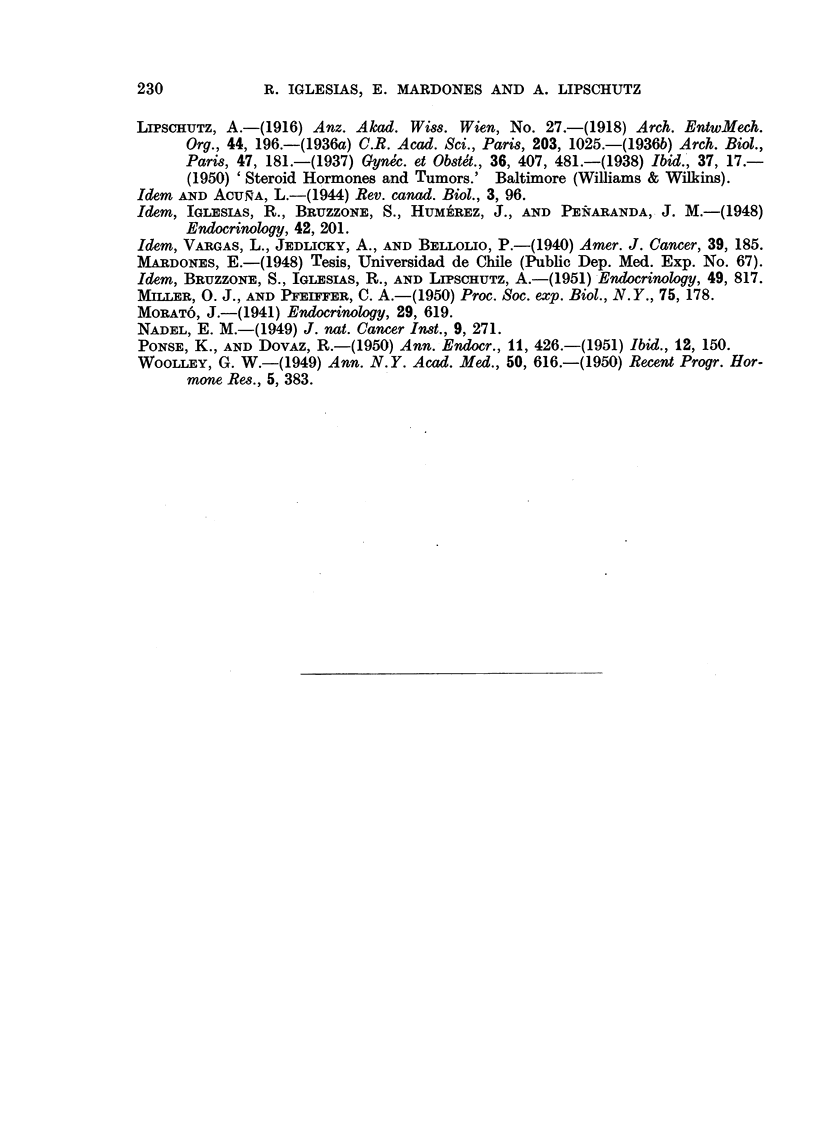# The Hormonal Background of Ovarian Experimental Tumourigenesis in the Guinea-Pig

**DOI:** 10.1038/bjc.1953.20

**Published:** 1953-06

**Authors:** R. Iglesias, E. Mardones, A. Lipschutz

## Abstract

**Images:**


### THE HORMONAL BACKGROUND OF OVARIAN EXPERIMENTAL TUMOURIGENESIS IN THE GUINEA-PIG.

R. IGLESIAS, E. MARDONES AND A. LIPSCHUTZ.

*From Departamento de Medicina Experimental, Servicio Nacional de Salud, Avenida Irarrázaval 849, Santiago de Chile.*

Received for publication April 2, 1953.

THE tumours which appear in the intrasplenic ovarian graft in castrated rats, mice, guinea-pigs or rabbits apparently are due to the uncontrolled gonadotrophic activity of the prehypophysis, the ovarian steroid hormones which normally control hypophysial activity being inactivated during their passage through the liver before reaching the general circulation (Iglesias, Mardones and Lipschutz, 1953). The most striking proof in favour of this explanation is given by the administration of ovarian hormones. When oestrogen is administered the intrasplenic ovarian graft in the castrated guinea-pig offers the aspect of a normal ovary ; hemorrhagic follicles and irregular luteal cords or nodules so characteristic of the intrasplenic graft in the guinea-pig fail to appear and seemingly normal corpora lutea are present (Lipschutz, Iglesias, Bruzzone, Humérez and Peñaranda, 1948). On the other hand, corpora lutea, but also luteomata, are counteracted when progesterone is administered for several months (Mardones, 1948 ; Iglesias, Lipschutz and Mardones, 1950 ; Mardones, Bruzzone, Iglesias and Lipschutz, 1951). In mice the ovarian tumours arising in intrasplenic grafts can be prevented when oestrogen or androgen is given (Li and Gardner, 1949).

It seemed, at first sight, rather contradictory when it was found that inactivation of oestrogen under these experimental conditions was in general incomplete, as evidenced by the increase of the weight of the uterus and vagina (Bernstorf, 1951), and that granulosa-cell tumours and luteomata may develop in the intrasplenic ovarian graft in castrated rats with persistent oestrus (4 cases described by Lacour, Oberling and Guérin, 1951).

In the present paper the question is examined whether ovarian tumourigenesis in experiments of long duration in the guinea-pig presupposes suppression of oestrogenic action in the body. We shall be able to show (1) that this is not the case, and (2) that the hormonal imbalance from which ovarian tumourigenesis under the given experimental conditions derives can, and must, be expressed in quantitative terms.

For the purpose of this study the secretory activity of 15 intrasplenic grafts, as listed in Table I of our previous paper (Iglesias, Mardones and Lipschutz, 1953) has been checked by the histological examination of the vaginal mucosa, the uterus and the mammary glands. The material included 8 grafts with luteomata and 7 without luteomata. A series of 9 experiments is added in which after castration and intrasplenic grafting minute quantities of oestrogen were administered during 26 to 27 months.

### *Secretory Activity of the Experimental Luteoma.*

A. *" Exclusive " luteoma.*

The relevance of the problem can best be exemplified by the condition as found in a case of exclusive luteoma, i.e., when Graafian follicles are absent or almost absent and the whole graft consists of luteal tissue without individual corpora lutea being present (Fig. 1).

This luteoma, together with some others pictured in our previous paper, was one of the most convincing examples of an ovarian tumour induced in the guinea-pig by the method of intrasplenic grafting. The animal was necropsied 997 days after castration and grafting. The nipples grew from 0·5 to 5 mm. in length. The mammary glands were as developed as in the second half of pregnancy ; the glandular acini were distended by secretion (Fig. 4). The vaginal mucosa was in the " transitional " state : several layers of the proliferated basal epithelium with superposed layers of large vacuolized cells (Fig. 5). This condition indicates oestrogenic action not completely counteracted by progesterone as in pregnancy. The uterine weight was 2·2 g., i.e., more than double that of the normal. There was a very pronounced cystic glandular hyperplasia of the endometrium with formation of polyps (Fig. 6) ; at different places there was mucous metaplasia of the endometrium or of the glands (Fig. 6 and 7).

It is evident that both oestrogen (the condition of the mammary glands and of the uterus) and progesterone (the condition of the vaginal mucosa) reached the general circulation. But androgen also was circulating in the body, as evidenced by the growth of the corpora cavernosa of the clitoris and of the horny styles ; this kind of masculinization, known for a long time (Lipschutz, 1916, 1918) can nowadays be easily produced by the administration of various androgens. It would be idle to discuss whether the androgen was produced in this case by the adrenals or the luteomatous ovary, or in which part of the latter oestrogen and progesterone were produced. The microscopical picture of the luteoma makes it in any case very probable that it was in a state of active secretion (Fig. 3).

An escape of oestrogen into the general circulation as in the animal described, is not a necessary accompaniment of each exclusive luteoma, as shown by the fact that such an escape was absent in another animal in which the microscopical condition of the luteomatous tissue was identical with that of Fig. 2 and 3. In the animal here described in detail the escape was facilitated by adhesions between the spleen and abdominal wall.

Our point is that the control of the gonadotrophic activity of the hypophysis was not completed, as shown by the presence of an haemorrhagic follicle (Fig. 1) and by the evolution of a luteoma so to say to the peak—though ovarian hormones were present in the general circulation, and though the quantity of circulating oestrogen was sufficient to produce vaginal, uterine and mammary development, and even an abnormal condition of the endometrium which presupposes protracted oestrogenic action.

B. *The condition of the vaginal and uterine mucosa in animals with and without luteoma.*

The findings as exemplified by the above description are fully corroborated by the results summarized in Table I. In no less than 5 out of the 8 animals with luteoma the vaginal mucosa evidenced action of both progesterone and oestrogen (" pregnancy " and " transitional " ; see for the latter Fig. 5). It was the same with the uterine mucosa. Indeed, the high columnar glandular epithelium as characteristic of oestrogenic action (Fig. 6 and 7) and even metaplasia—which is obtained in the guinea-pig by the prolonged administration of small quantities of oestrogen (Lipschutz, Vargas, Jedlicky and Bellolio, 1940)—were present in 2 animals only. In the remaining 6 animals both the condition of the uterine epithelium and of the myometrium were as in castrated animals. There was, however, one notable feature in the uterus never found in castrated animals : the presence of numerous small glandular cysts lined with an epithelium poor in protoplasm. This condition denounces prolonged oestrogenic action at a level sufficient to produce cystic glandular hyperplasia ; but evidently this level was not maintained indefinitely (Lipschutz and Acuña, 1944 ; Lipschutz, 1950, p. 203). The presence of ovarian hormones in the general circulation at a level allowing for the transformation of the vaginal mucosa and of the uterine epithelium, even with the production of cystic glandular hyperplasia and metaplasia, was not concomitant with the control of the gonadotrophic activity of the hypophysis as evidenced by the appearance of haemorrhagic follicles.

It was the same in the group without luteoma (Table I).

TABLE I.—*Condition of the Vaginal and Uterine Mucosa in* 15 *Animals With or Without Luteoma* 612 *to* 1093 *days after Intrasplenic Grafting.*

| Group. | Total. | With haemorrhagic follicles. | Vaginal mucosa. | | | | Uterine mucosa. | | | Cystic. |
|---|---|---|---|---|---|---|---|---|---|---|
| | | | Castration. | Pregnancy*. | Transitional**. | Oestrus. | Castration. | High epithelium. | Metaplasia. | |
| With luteoma | 8 | 3 | 3 | 3 | 2 | 0 | 6 | 2 | 2 | 3 |
| Without luteoma | 7 | 4 | 2 | 0 | 4 | 1 | 6 | 1 | 0 | 2 |

* Pregnancy = several layers of clear vacuolized epithelia.

** Transition = several layers of proliferated cells of the basal epithelium beneath the vacuolized layers as in Fig. 5, indicating oestrogenic action not completely counteracted by progesterone as in pregnancy.

Whereas the above findings show that the ovarian hormones reaching the general circulation from the luteomatous or non-luteomatous intrasplenic graft fail to control the gonadotrophic activity of the hypophysis, they do not show that the hypophysis is freed from any control. As evidenced by various new observations, we must drop the idea held originally both by Biskind and by ourselves as to the gonadotrophic activity of the hypophysis in experiments with intrasplenic ovarian grafts in castrated females : contrary to what we thought before, the hypophysis is, under these experimental conditions, not identical to a castrate hypophysis. Thus, the content of gonadotrophic hormones in the hypophysis of animals with grafts is considerably less than in the castrate hypophysis (Jungck, Heller and Nelson, 1947). Indeed this non-identity may be due to a difference, not of production, but only of release of gonadotrophins in castrated and grafted animals (Miller and Pfeiffer, 1950) ; but non-identity seems to be a fact. Castration cells are absent in the hypophysis of the grafted rat, in any case in advanced stages of ovarian tumourigenesis (Lacour and Guérin, 1951).

However, as compared to a normal hypophysis, that of a castrated animal with an intrasplenic ovarian graft is functionally impaired.

*Differential Tumourigenic Threshold Concentrations of Oestrogen.*

The problem of differential levels of oestrogen by which control of the vaginal or uterine mucosa and of the hypophysial gonadotrophic activity is achieved is intimately related to the problem of the differential tumourigenic threshold concentrations of oestrogen. The latter problem has already been discussed elsewhere (Lipschutz, 1950, p. 45). Its relevance with reference to ovarian tumourigenesis due to an impairment of the gonadotrophic function of the hypophysis is best exemplified by the description of an experiment in which the available oestrogen was not only that occasionally escaping from the luteomatous or non-luteomatous graft, but also oestrogen administered, though in quantities not sufficient to obtain complete control of the hypophysis.

It is known that quantities of oestrogen which are absorbed from a subcutaneously implanted pellet containing but 1 per cent of oestradiol and 99 per cent of cholesterol are not always sufficient, even when acting during 6 weeks, to suppress haemorrhagic follicles in the intrasplenic graft, though they may produce in the same animal abnormal increase of uterine weight, cystic glandular hyperplasia and polyposis (Barahona, Bruzzone and Lipschutz, 1950). How will the hypophysis behave when exposed to the action of these minute quantities of oestrogen in experiments lasting more than 2 years ?

A group of 9 guinea-pigs was castrated and one ovary was grafted into the spleen ; sixteen days later a pellet of 23 to 30 mg. containing 1 per cent oestradiol was implanted subcutaneously. Necropsy was made 795 to 828 days after grafting. The graft was present in all the animals of the group. The size of the graft was variable ; in some animals it reached about 1 c.c., but in the majority of the animals it was smaller than that. Haemorrhagic follicles were found in 7 out of 9 animals. There were large follicular cysts, large corpora lutea, nodules of luteal cells ; but in none of the 9 animals could the condition be considered as luteomatous, comparable to what has been described with ovarian grafts in experiments of a similarly long duration but without the continuous action of oestradiol (Iglesias, Mardones and Lipschutz, 1953). In some grafts Wolffian cysts of a variable size were found. In one animal, necropsied at 828 days after grafting, there was an adenofibroma of the Brenner type, about 1 mm. in diameter, dense fibrous tissue prevailing (Fig. 8 and 9).

As evident from the above description the small quantities of oestradiol absorbed in the course of the experiment apparently exercised in time some control of the gonadotrophic activity of the hypophysis in such a way that no clear-cut luteoma appeared in this group. But the quantities absorbed were not sufficient for a complete control of this hypophysial activity : they were not able to reduce it to normality, as shown by the presence of haemorrhagic follicles in most of the animals of the group.

There was in this group control of the vaginal mucosa and of uterine growth. In one animal the uterine weight was only 0·6 g. ; but in the remaining animals a weight of 0·8 to 1·2 g. and in one case of 4 g. was reached. The uterine epithelium was in several animals columnar; in many cases glandular cysts were present. Of an especial interest as to the uterine condition was the animal in which the uterine weight was highest when necropsied 828 days after grafting.

A tumour several mm. in diameter was found on the parametrial border of the left uterine horn (Fig. 10). The tumour was a fibroid, or rather a desmoid, of the kind one finds in animals with the very prolonged action of oestrogen. With such small quantities of oestrogen fibroids have never been seen before even in experiments of long duration (Lipschutz, 1950, p. 44). One may venture that in the present case fibromatogenic action was due to the combined quantities of oestrogen both absorbed from the 1 per cent pellet and produced by the graft.

There was in this animal also a cystic glandular hyperplasia with proliferation of glands and invasion of the myometrium; the glands reached the thickened serosa; many of these glands were distended; some were in immediate contact with the desmoid described above (Fig. 10 to 13). Invasive proliferation of uterine glands induced by quantities of oestrogen so small as that absorbed from 1 per cent pellets in experiments of long duration has been already described (Lipschutz, 1950). Indeed, with these 1 per cent pellets the incidence is not great; in the present group of 9 animals it occurred but once. In another new experiment lasting 757 to 875 days, without intrasplenic ovarian grafts, it occurred likewise but once in a group of 10 animals. On the contrary in the former series already referred to the incidence was of 11 among 14 animals in experiments lasting 539 to 870 days.

In the animal under discussion the vaginal mucosa was that of oestrus; there were many layers of epithelial cells with partial cornification. Epithelial proliferation was probably atypical; there were ingrowths into the submucosa, some with horny pearls (Fig. 15). The condition of the vaginal mucosa denotes that oestrogen was active till the end of the experiment, i.e., the 260 $\mu$g. of oestradiol originally present in the implanted pellet of 26 mg. were not yet absorbed 812 days later—a statement repeatedly made in this Department (Barahona, Bruzzone and Lipschutz, 1950; unpublished work of Iglesias and Mardones).

The results here described leave no doubt that minute quantities of oestrogen, which when circulating continuously in the body for as long as 27 months are liable to elicit epithelial and conjunctive tumourigenesis, may fail to control the hypophysis, as evidenced by the presence of haemorrhagic follicles in the intrasplenic graft. We shall see in the next section that knowledge of the comparative tumourigenic threshold concentrations of oestrogen and of the level at which oestrogen controls the gonadotrophic function of the hypophysis apparently is fundamental for the true understanding of tumourigenesis due to hormonal imbalance.

### DISCUSSION.

Our knowledge on tumourigenesis in ovarian intrasplenic grafts in the guinea-pig may be summarized in the scheme given in Table II.

Ovarian tumourigenesis in the graft is produced when oestrogen is absent in the general circulation (Threshold 0); but ovarian tumourigenesis is elicited also when the quantity of oestrogen, compared to normality, is so diminished that complete control of the gonadotrophic function of the hypophysis cannot be achieved (Threshold 1), though this level of oestrogen allows for a considerable hyperplasia and metaplasia of the uterine epithelia, and for polyposis. At a

TABLE II.—*Differential Thresholds of Oestrogen Concentration, Degrees of Hypophysial Control and Experimental Tumourigenesis in the Guinea-pig.*

| Differential thresholds of oestrogen concentration. | Uterine epithelia. | Conjunctive tumourigenesis of abdominal serosa. | Control of gonadotrophic function of hypophysis. | Intrasplenic ovarian graft. | | | Examples. |
|---|---|---|---|---|---|---|---|
| | | | | Luteoma. | "Brenner". | Haemorrhagic follicles. | |
| ↑ 3 | Invasive proliferation of uterine glands | + | Incomplete but greater than at 1 | 0 | + | + | Fig. 8–15. |
| 2 | Invasive proliferation of uterine glands | 0 | Incomplete but greater than at 1 | 0 | + | + | Fig. 8–15. |
| 1 | Cystic glandular hyperplasia, metaplasia, polyposis | 0 | None, or incomplete | + | + | + | Fig. 1–7. |
| 0 | Castrate | 0 | None | + | + | + | Table I, first column of "vaginal mucosa." |

higher level of oestrogen in the general circulation (Threshold 2) there may be invasive proliferation of the uterine glands ; the oestrogen concentration may be sufficient to control the gonadotrophic function of the hypophysis in so far as luteoma is absent. However, this does not mean complete control of the hypophysis, since the Brenner type of tumour and haemorrhagic follicles may still be found in the graft. They may occur even at Level 3, which is as high as to allow exceptionally for conjunctive tumourigenesis (Fig. 10).

Indeed, when referring to an impairment of the gonadotrophic function of the hypophysis due to a decrease of the level of oestrogen in the general circulation one simplifies matters very considerably. What is the normal sexual cycle is the outcome of an interplay of *three* gonadotrophic hormones—FSH, LH and LTH (LTH is the luteotrophic hormone, or prolactin) whose production and release are interfered with by *two* ovarian hormones, oestrogen and progesterone, and in this interplay besides the gonads and the hypophysis also the hypothalamus is implicated (Harris, 1950 ; Harris and Jacobsohn, 1952). These experimental data are a warning against the tendency to explain pathological conditions of the ovary in women by the failure of *one* hormone—as is so often done ; and, on the other hand, these data must be borne in mind also when trying to explain experimental ovarian tumourigenesis induced by intrasplenic transplantation in castrated animals. The ovarian tumours under these experimental conditions result from an irregular interplay of no less than *five* hormones !

In castrated rats receiving daily injections of oestrogen during 45 days control of the gonadotrophic activity of the hypophysis can be achieved by quantities of oestrogen which are not yet sufficient to promote uterine growth (Byrnes, Meyer and Finerty, 1951 ; Greep and Jones, 1950). We are unable to explain why the hypophysis behaves in experiments with intrasplenic grafts in a manner so different from what has been observed in rats under the related experimental conditions. It is not a species difference ; what we have seen in guinea-pigs is coincident with what other authorities have described in rats (Lacour, Oberling, and Guérin, 1951 ; Lacour and Guérin, 1951) and mice (Bernstorf, 1951). One may raise the question whether some irreversible hypophysial change takes place during those 2 to 3 weeks which are needed for the vascularisation of the graft and for recuperation of hormone production.

We have referred above to the three gonadotrophic hormones of the hypophysis. But the possibility must be considered that also the somatotrophic or some other hypophysial organotrophic hormone is partaking in establishing tumoural growth in the ovary ; this question has been discussed especially with reference to tumoural growth of Wolffian structures (Iglesias, Mardones, Bruzzone and Lipschutz, 1953).

The statement that ovarian tumourigenesis does not presuppose a complete suppression of the steroid control of the hypophysis but is the outcome of an irregular interplay of ovarian and hypophysial hormones may at the first sight seem trivial and vague. However, not only is it in agreement with the experimental facts related in the present paper and summarized in Table II, but it opens also the way to a better understanding of certain experimental and spontaneous types of tumourigenesis.

Let us refer first to atypical and tumoural growth of the ovary and of the uterine epithelium as induced by " ovarian fragmentation " (Lipschutz, 1936*a*, 1937, 1938 ; Morató, 1941) ; and to tumoural growth outside the genital tract induced by the same method (Nadel, 1949 ; Ponse and Dovaz, 1950, 1951 ; Bruzzone, 1950). This kind of tumourigenesis has been attributed long since to an hormonal imbalance : there was an increased content of gonadotrophic hormones in the hypophysis (Lipschutz, 1936*b*), there were follicular and luteic cysts, sometimes haemorrhagic ones, protracted oestrus and uterine bleeding but also masculinization of the clitoris (Lipschutz, 1937, 1938). The hormonal imbalance may last for as long as 3 years, and sometimes even more, without normality being again attained. Whereas it was evident, almost from the beginning of this phase of our work, that the sequels of ovarian fragmentation, i.e., reducing the mass of two ovaries to a small ovarian fragment, or remnant, *in situ*, were due to an overthrow of the normal ovarian-hypophysial relationship, we were unable to understand why this hormonal imbalance is in some cases irreversibly maintained. Why the oestrogen produced by the ovarian fragment, and sometimes even in quantities sufficient to produce epithelial or conjunctive tumorigenesis, fails in gaining control over the hypophysis ? And even still very recently we had to confess that " as to this we are to-day not more advanced than we were 12 years ago " (Lipschutz, 1950, p. 220). Nowadays one may tentatively suggest that ovarian fragmentation creates quantitative and timing conditions of oestrogen production which are still in the limits of threshold 3 of our Table II. This refers also to other kinds of operative interferences on the ovary (Lipschutz, 1950, pp. 191, 220 ; Bielschowsky and Hall, 1951*b*, 1952). Blood follicles may appear occasionally in intrarenal grafts (Lipschutz, 1950, p. 178). Even neoplastic changes may occur, though only exceptionally, in a subcutaneously grafted ovary (Bielschowsky, 1951*a*).

Our results as summarized in Table II would also explain observations with suprarenal tumours induced in certain strains of mice by castration (Woolley, 1949, 1950), the corticotrophic activity of the hypophysis being evidently freed from the control by ovarian hormones. Later on these tumours themselves produce oestrogen by which mammary carcinoma is induced, but without the control of the corticotrophic activity being re-established. Indeed, our Table II does not consider the eventuality that the maintenance of the hormonal imbalance may be due also to the lack of other steroid hormones, besides oestrogen, and in the first place progesterone.

Our results may be applied also to tumourigenesis in women in menopause. The urine in the menopause contains hypophysial gonadotrophic hormones; but uterine fibroids begin to regress. As to these two phenomena, the condition may be compared to that taking place in the guinea-pig with the Threshold 2 in Table II: the oestrogen produced is not sufficient for a complete control of the hypophysis and for conjunctive tumourigenesis. However, the menopause is the preferential age for atypical and neoplastic growth of the uterine epithelia which can be elicited experimentally with Threshold 2 of oestrogen. Indeed,

---

### EXPLANATION OF PLATES.

*Fig.* 1 *to* 7 *are from the same animal*; *see Fig.* 1.

FIG. 1.—" Exclusive " luteoma in intrasplenic ovarian graft; 997 days (CXXVI, 191). No individual corpora lutea. In this animal there was secretion both of oestrogen and androgen. An haemorrhagic follicle on the right. × 5.

FIG. 2.—" Compact " luteal tissue at the periphery of the luteoma. × 285.

FIG. 3.—Central part of luteoma with " loose " luteal tissue. Signs of secretion. × 285.

FIG. 4.—Mammary gland. Acini with secretion. × 67.

FIG. 5.—Vaginal mucosa. Proliferation of basal layers indicating oestrogenic action. × 70.

FIG. 6.—Uterus. Cystic glandular hyperplasia. Polyps. Metaplasia of glandular epithelium. × 15.

FIG. 7.—The same as Fig. 6. Highly developed endometrium. Metaplasia. × 67.

*Fig.* 8 *to* 15 *are from the same animal*; *see Fig.* 8.

FIG. 8.—Intrasplenic ovarian graft, non-luteomatous; 828 days after transplantation; 812 days of action of oestrogen absorbed from subcutaneous tablet containing but 1 per cent of oestradiol (CLI., 231). Various corpora lutea and a haemorrhagic follicle. There were various other haemorrhagic follicles. Large Graafian follicle to the right. Large cystic Wolffian structure beneath. Fibroadenomatous nodule at ↓, partly contacting with the spleen. × 15.

FIG. 9.—Detail of the fibroadenomatous nodule. Two tubular structures merging in fibrous tissue which prevails in this nodule. × 300.

FIG. 10.—Uterine horn with fibroid on the side of the parametrium. Uterine cavity almost disappeared. Very pronounced cystic glandular hyperplasia. Penetration of glands into the irregularly hypertrophied myometrium. Cystic glands outside the uterus contacting with the fibroid. × 5.

FIG. 11.—Uterine glands immediately beneath the thickened serosa; one of the glands distended. At * proliferation of the endothelium of the serosa. × 70.

FIG. 12.—Group of crowded glands immediately beneath the somewhat thickened serosa. × 300.

FIG. 13.—Distended glands in the myometrium, beneath the thickened serosa. × 70.

FIG. 14.—Detail of Fig. 11; the glands at the extreme right. Several mitoses (?). × 300.

FIG. 15.—Vaginal mucosa. Oestrus; cornification. Apparently disorderly proliferation of the epithelium. Horny pearls. × 70.

Iglesias, Mardones and Lipschutz.

Iglesias, Mardones and Lipschutz.

here again our scheme may be incomplete in so far as the controlling role of progesterone has not been considered.

### SUMMARY.

Control of the gonadotrophic activity of the hypophysis was not achieved and evolution of luteoma in the intrasplenic graft of castrated guinea-pigs was not inhibited by an escape of oestrogen into the general circulation in quantities sufficient to produce proliferation and metaplasia of the uterine epithelium and polyposis.

Evolution of luteoma in the intrasplenic graft apparently was inhibited in a series of animals to which oestrogen was administered through absorption from a subcutaneous pellet containing but 1 per cent of oestrogen. However, in time —27 months—such a pellet may induce an invasive proliferation of the uterine glands and, exceptionally, also a uterine fibroid. But even in these animals without luteoma entire control of the gonadotrophic activity of the hypophysis was not achieved, as evidenced by the presence of haemorrhagic follicles in most of the animals and of a fibroadenomatous nodule of the Brenner type in one of these animals.

The variable occurrence of oestrogen-induced epithelial and conjunctive tumourigenesis of the genital tract, in animals in which the oestrogen fails to control hypophysial gonadotrophic activity paramount for protection against ovarian tumourigenesis, can be understood only in quantitative terms, as expressed in the concept of " differential tumourigenic threshold concentrations of oestrogen ".

The concept of differential tumourigenic threshold concentrations of oestrogen as based on experimental lines opens an understanding for the fact, so contradictory at first sight, that in women in menopause when uterine fibroids involute, atypical growth of uterine epithelia known to be dependent on oestrogen can still be stimulated.

### REFERENCES.

Barahona, M., Bruzzone, S., and Lipschutz, A.—(1950) *Endocrinology*, **46**, 407.
Bernstorf, E. C.—(1951) *Ibid.*, **49**, 302.
Bielschowsky, F.—(1951*a*) *Rep. Brit. Emp. Cancer Campgn.*, **29**, 242.
*Idem* and Hall, W. H.—(1951*b*) *Brit. J. Cancer*, **5**, 331.—(1952) *Proc. Univ. Otago med. Sch.*, **29**, No. 3.
Bruzzone, S.—(1950) *Rev. Med. Aliment., Santiago*, **9**, 11.
Byrnes, W. W., Meyer, R. K., and Finerty, J. C.—(1951) *Amer. J. Physiol.*, **164**, 26.
Greep, R. O., and Jones, I. C.—(1950) *Recent Progr. Hormone Res.*, **5**, 197.
Harris, G. W.—(1950) *J. Physiol.*, **111**, 347.
*Idem* and Jacobsohn, D.—(1952) *Proc. Roy. Soc.*, B, **139**, 263.
Iglesias, R., Lipschutz, A., and Mardones, E.—(1950) *J. Endocrinol.*, **6**, 363.
*Idem*, Mardones, E., and Lipschutz, A.—(1953) *Brit. J. Cancer*, **7**, 214.
*Iidem*, Bruzzone, S., and Lipschutz, A.—(1953) *Arch. Anat. micr. Morph. exp.* (In press.)
Jungck, E. C., Heller, C. G., and Nelson, W. O.—(1947) *Proc. Soc. exp. Biol. N.Y.* **65**, 148.
Lacour, F., and Guérin, M.—(1951) *Bull. Ass. franç Étude Cancer*, **38**, 423.
*Idem*, Oberling, Ch., and Guérin, M.—(1951) *Ibid.*, **38**, 128.
Li, M. H., and Gardner, W. U.—(1949) *Cancer Res.*, **9**, 39.

LIPSCHUTZ, A.—(1916) *Anz. Akad. Wiss. Wien*, No. 27.—(1918) *Arch. EntwMech. Org.*, **44**, 196.—(1936*a*) *C.R. Acad. Sci., Paris*, **203**, 1025.—(1936*b*) *Arch. Biol., Paris*, **47**, 181.—(1937) *Gynéc. et Obstét.*, **36**, 407, 481.—(1938) *Ibid.*, **37**, 17.—(1950) ' Steroid Hormones and Tumors.' Baltimore (Williams & Wilkins).
*Idem* AND ACUÑA, L.—(1944) *Rev. canad. Biol.*, **3**, 96.
*Idem*, IGLESIAS, R., BRUZZONE, S., HUMÉREZ, J., AND PEÑARANDA, J. M.—(1948) *Endocrinology*, **42**, 201.
*Idem*, VARGAS, L., JEDLICKY, A., AND BELLOLIO, P.—(1940) *Amer. J. Cancer*, **39**, 185.
MARDONES, E.—(1948) Tesis, Universidad de Chile (Public Dep. Med. Exp. No. 67).
*Idem*, BRUZZONE, S., IGLESIAS, R., AND LIPSCHUTZ, A.—(1951) *Endocrinology*, **49**, 817.
MILLER, O. J., AND PFEIFFER, C. A.—(1950) *Proc. Soc. exp. Biol., N.Y.*, **75**, 178.
MORATÓ, J.—(1941) *Endocrinology*, **29**, 619.
NADEL, E. M.—(1949) *J. nat. Cancer Inst.*, **9**, 271.
PONSE, K., AND DOVAZ, R.—(1950) *Ann. Endocr.*, **11**, 426.—(1951) *Ibid.*, **12**, 150.
WOOLLEY, G. W.—(1949) *Ann. N.Y. Acad. Med.*, **50**, 616.—(1950) *Recent Progr. Hormone Res.*, **5**, 383.